# Neurodegenerative disorder and diffuse brain calcifications due to *FARSB* mutation in two siblings

**DOI:** 10.1002/ccr3.6195

**Published:** 2022-08-03

**Authors:** Parvaneh Karimzadeh, Sepideh Rezakhani, Mohammad Miryounesi, Sahar Alijanpour

**Affiliations:** ^1^ Pediatric Neurology, Pediatric Neurology Research Center, Mofid Children's Hospital Shahid Beheshti University of Medical Sciences Tehran Iran; ^2^ Medical Genetics, Genomic Research Center, Taleghani Hospital Shahid Beheshti University of Medical Sciences Tehran Iran

**Keywords:** aminoacyl‐tRNA synthetase, brain calcification, developmental delay, *FARSB*

## Abstract

Pathogenic mutations in the *FARSB* gene are associated with neurodevelopmental disorder involving the brain, liver, and lungs. We report genetic analysis of a family including two affected members with this disorder, which revealed a homozygous pathogenic missense variant, *FARSB*: NM_005687.4:c.853G > A:p.E285K in both affected patients. The parents were heterozygous for this variant.

## Graphical abstract

Early diagnosis of atypical cases of *FARSB* mutation in children with multisystem disorders provides the chance to imply novel treatments for them as well as carrier detection and prenatal diagnosis. Therefore, performing genetic test such as Whole Exome Sequencing could help us in this regard.

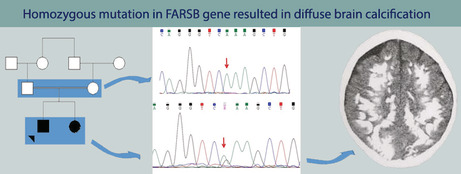

## 1 INTRODUCTION

Aminoacyl‐tRNA synthetases (ARSs or aaRSs), also called tRNA‐ligases, are mitochondrial and cytoplasmic enzymes that attach the appropriate amino acids onto their corresponding tRNA, a process that is required for the first step of protein synthesis. These enzymes are also engaged in the survival of the cell through regulating cellular signaling and metabolism.^1^ Therefore, dysfunction or absence of ARSs results in severe or lethal conditions. To date, 31 ARS loci are known to be related to dominant and recessive human disease phenotypes.^2^, ^3^, ^4^

The human PheRS (phenylalanyl‐tRNA synthetase) is an ARS that charges tRNA with phenylalanine in the cytoplasm and is made of two subunits coded by *FARSB* gene and two subunits coded by *FARSA* gene.^5^, ^6^ Mutation variants in *FARSA* or *FARSB* implicated in human disease are very rare.^7^ Here we report two siblings presented to the pediatric neurology clinic with neurodegenerative disorder and diffuse brain calcifications with homozygous variant *FARSB* gene.

## 2 CASE PRESENTATION

### 2.1 Patients and clinical data

**FIGURE 1:**
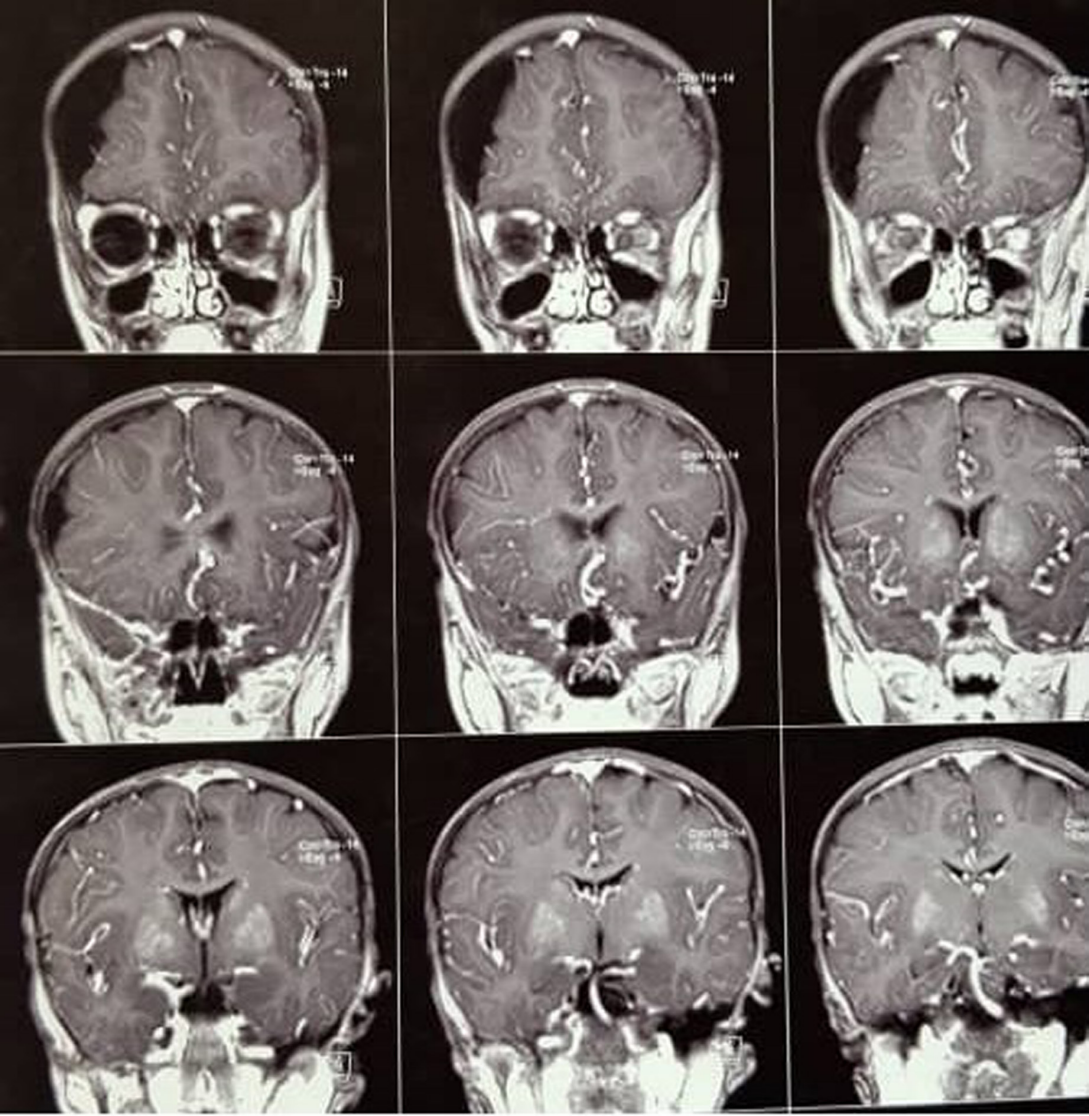
Coronal planes of brain MRI of the proband case demonstrate symmetrical high signal intensities in putamen and a subdural effusion in right side

**FIGURE 2:**
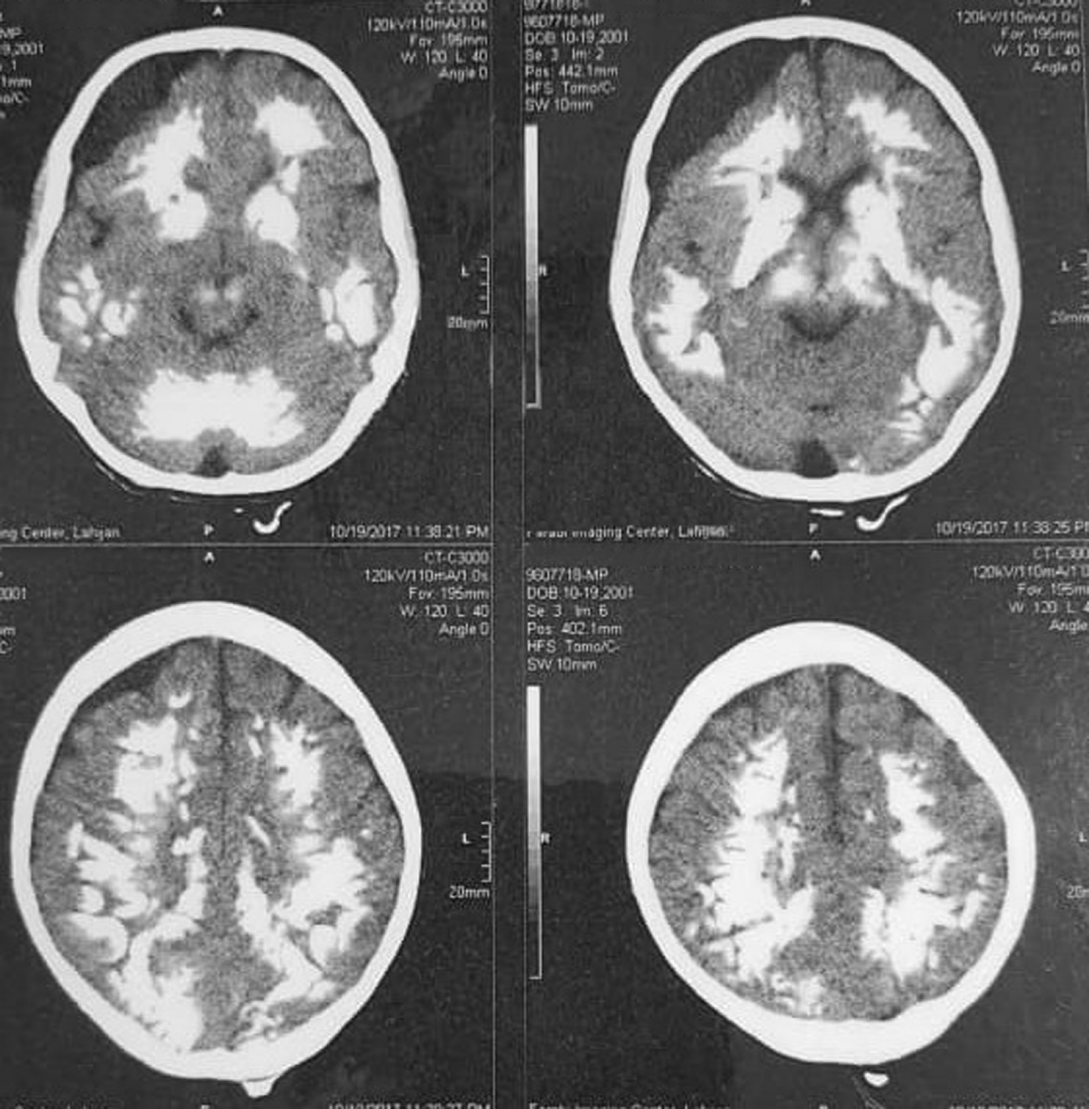
Axial planes of brain CT scan of the proband case demonstrate diffuse symmetrical calcifications together with subdural effusion in right side

The patients are two siblings born to consanguineous parents (first cousins). The first case is an 18‐year‐old male with a normal prenatal and birth history. Growth and developmental history was normal except for being underweight and a delay in completing toilet training. He seems normal mentally with normal IQ and is now graduating from high school. From the age of 4 to 8 years, he had been treated with multiple antiseizure drugs due to refractory focal seizures. He also complained of intermittent headaches at that time. Brain MRI (Figure 1) revealed subdural effusion along with subdural hematoma, and brain CT‐scan (Figure 2) demonstrated bilateral diffuse parenchymal calcifications. From the age of 8, seizures and headaches were controlled but he developed ataxia, hemiparesis, and an abnormal gait due to dystonia. He had dystonia in all limbs and increased (3+) deep tendon reflexes (DTR) in both knees.

**FIGURE 3:**
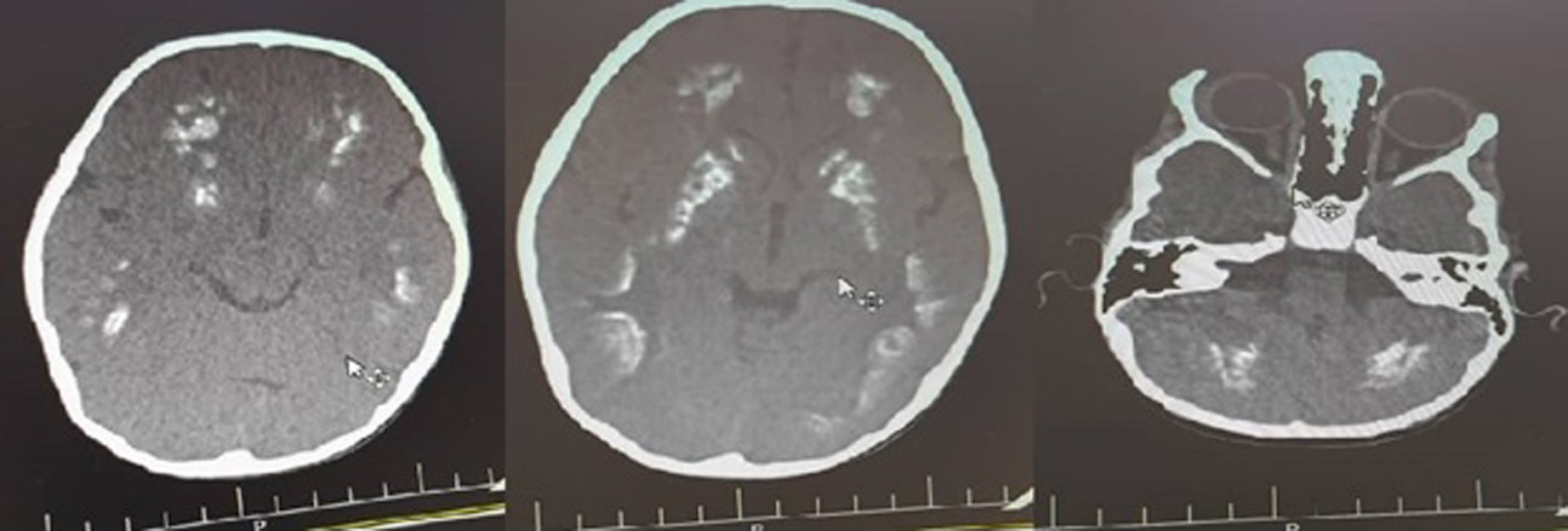
Axial planes of brain CT‐scan of the younger sibling demonstrate diffuse symmetrical calcifications

The younger sibling is a 4‐year‐old female with normal prenatal, birth, and neurodevelopmental history who has recently experienced falling episodes followed by brief lethargy. She developed weakness of the right upper limb after one of the falling episodes. She had right lower limb dystonia, dystonic gait, and increased (3+) DTR on physical examination. Brain CT scan demonstrated diffuse calcification of the parenchyma (Figure 3). EEG was abnormal and primidone was initiated. Levodopa‐carbidopa was also prescribed.

The first patient had a history of lung surgery due to pneumothorax and bullous emphysema. He also had reticular opacities in both parahilar regions and right upper zones on chest radiography but no pulmonary calcification was noted. The sister had no lung abnormality based on the imaging and history. Ultrasound imaging of the hepatobiliary systems was normal in both patients.

Considering the calcifications in neuroimaging of the proband case together with consanguinity of the parents and similar findings in the sibling, as well as ruling out hypoparathyroidism, calcium ion metabolism disorder, metabolic and mitochondrial disorders, FAHR disease was the most probable diagnosis at that time. However, there was no autosomal dominant pattern and so autosomal recessive disorder was brought in the mind and whole‐exome sequencing (WES) was performed to look for other possible genetic disorder, which revealed *FARSB* mutation.

### 2.2 Genetic analysis

#### 2.2.1 Genomic DNA extraction

To perform molecular study, Genomic DNA (gDNA) of the patients and their parents were extracted from the peripheral blood samples using the standard salting‐out method.^8^

#### 2.2.2 Whole‐Exome Sequencing (WES)

It was performed in the proband using custom‐designed Nimblegen chip‐capturing array. Nearly 60 Mb of the targeted region on consensus coding sequences enriched from fragmented genomic DNA of the proband by around 758,086 probes designed for the human genome (Agilent SureSelectXT2 V7 exome). Then sequencing was performed on Illumina HiSeq4000 platform (Illumina). Paired‐end sequence reads were aligned to the human reference genome (UCSC hg19/ GRCh37) using Burrows‐Wheeler Aligner (BWA). Variant calling was run using SAMTools and Genome Analysis Toolkit (GATK v 3.7).^9^, ^10^, ^11^ Moreover, ANNOVAR software annotated and filtered the variants. To further filtrations, all pathogenic variants described in HGMD®, as well as variants with minor allele frequency (MAF) less than 0.01% measured against gnomAD, ExAC, 1000Genome projects, dbSNP138, ESP6500, NHBL Exome Variant Server (EVS), and Iranome. The functional effects of the variants were appraised in silico by predictor tools including Polyphen2, SIFT, MutationTaster, and CADD software. Finally, sequencing result was also filtered based on inheritance pattern, variant type, and phenotype.

#### 2.2.3 Sanger sequencing

Sanger sequencing was performed in the siblings and their parents to confirm and segregate the resulted variant. Amplifications were done with specific primers targeting the variant loci.

#### 2.2.4 Mutation analysis

**FIGURE 4:**
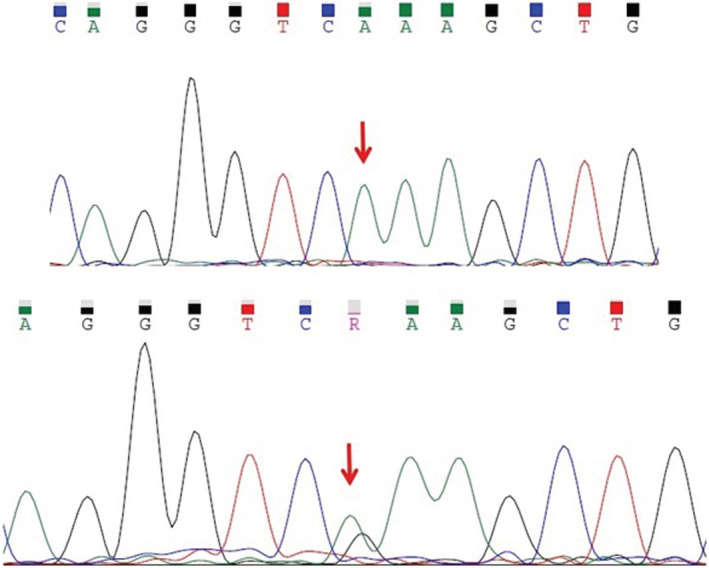
Sanger sequencing of all the family members for *FARSB* gene: as seen, both siblings have homozygous mutation and both parents have heterozygous state in this regard

The affected members of this family are homozygous for a pathogenic missense variant (NM_005687.4:c.853G > A:p.E285K) in *FARSB* gene. Their parents are heterozygous for this variant. The frequency of this variant in databases such as gnomAD, ExAC, Iranome, and 1000 Genome was zero. According to ACMG guidelines, this variant was classified as pathogenic. Figure 4 shows their Sanger sequencing pattern.

## 3 DISCUSSION

Aminoacyl‐tRNA synthetases (ARSs or aaRSs) conjugate specific aminoacids to the relevant tRNAs. This is sometimes called “charging” or “loading” the tRNA with an aminoacid. This process is an important and critical step in protein synthesis. Mutations in ARSs cause a variety of disorders with clinical picture determined by the location of the particular protein within the mitochondria, cytoplasm, or both. The human nuclear genome contains 37 ARS‐encoding loci. They encode 17 enzymes required for tRNA charging in the mitochondria, 17 enzymes required for tRNA charging in the cytoplasm, and three enzymes that charge tRNA in both cellular parts.^12^, ^13^ Phenylalanyl‐tRNA synthetase (PheRs) is a cytoplasmic aminoacyl‐tRNA synthetase and is formed by two *FARSA* and two *FARSB* subunits. *FARSA* and *FARSB* dimers assemble to form phenylalanyl‐tRNA synthetase complex. PheRs dysfunction results in translation defect, which causes failure in protein supply for organ tissues. High protein demand tissues such as the liver, lung, brain, and muscle are particularly affected by this genetic disorder.^14^

In general, *FARSA*‐ and *FARSB*‐related diseases are closely related in clinical presentations. The general manifestations include hypotonia, growth retardation, brain and liver involvement, and interstitial lung disease. Pulmonary involvement in *FARSB*‐related disease is reported to have variable onset and severity, and interstitial lung disease has been the most common presentation and mortality cause in these patients.^7^, ^15^ Pulmonary involvement as pneumothorax and bullous emphysema was also observed in the older sibling of our cases.

All reported ARSs deficiencies lead to extensive brain lesions or sensory organ deficits. In the *FARSB*‐related disease, brain calcifications were the most frequent findings followed by leukoencephalopathy and cerebral volume loss (as a result of incomplete closure of Sylvian fissure or hydrocephalus).^15^

Antonellis et al.^7^ described a compound heterozygous case for *FARSB* variants: p.Thr256Met and p.His496Lysfs*14. A severe depletion of both *FARSB* and *FARSA* protein levels was detected in fibroblast cells of the patient. The clinical presentation of these crucial mutations was a lethal multisystem developmental phenotype in their case; including intrauterine growth restriction and oligohydramnios, failure to thrive, developmental delay, abnormal face and genitalia, hydrocephalus, respiratory distress and diffuse patchy airspace opacities on chest radiograph, hepatocellular and renal disease, and multiple metabolic problems. The patient ultimately passed away at 32 months.

Since null variants in tRNA synthetase genes lead to severe multisystem dysfunctions, which are considered lethal, none of the reported patients with the *FARSB* disease had biallelic null loss‐of‐function variants^7^, ^16^ as in the 15‐year‐old case reported by Krenke et al.^15^ They concluded that at least one of the variants in the survived patients should have a residual activity.

In a review of the spectrum of *FARSA*‐ and *FARSB*‐associated diseases, Schuch et al described five unrelated patients from the European Management Platform for Interstitial Lung Disease (chILD‐EU) registry, with disease‐causing variants in *FARSA* and *FARSB*, all of which had biallelic variants and were identified due to chronic interstitial lung disease. Besides that, they had abnormalities in other organs/systems including the limbs, brain, liver, spleen, and gastrointestinal tract. They also demonstrated different severities of growth restriction.^17^ One of our patients had also pulmonary involvement and growth retardation besides the brain calcification.

The most relevant report for our cases is an interesting report from Zadjali et al about eight members of a large family in Oman with an autosomal recessive multisystem condition caused by the same variant of *FARSB* mutation as diagnosed in our patients (c.853G > A). Although the exact onset and severity were not consistent among all the affected cases, general findings were similar including growth restrictions, brain calcifications, and interstitial lung disease.^16^ The two siblings reported here also demonstrated the same phenotype slightly different in timing and intensity.

Presently, no standard therapeutic modality is available for ARS patients. Options are limited to symptomatic treatment such as brain ventricular shunts for hydrocephalus or whole lung lavage for transient relief of respiratory symptoms or antiseizure drugs for epileptic patients. Increasing the concentration of the amino acid as substrate appears to be a safe therapeutic solution, but this method should be utilized individually, and needs to be addressed in clinical trials.^17^, ^18^ Kok and colleagues described an individualized approach to treat the patients with corresponding amino acids. With lower related amino acid concentrations, fibroblast growth was intensely disturbed. Kok et al first performed genetic diagnoses and in vitro functional studies on fibroblasts obtained via skin biopsy, then treated the patients with related amino acids for instance L‐phenylalanine in *FARSB* mutation, and gained multisystem benefits in all patients.^18^ These findings can hopefully lead to further investigations for this approach with promising results.

Considering that currently there is no cure for this disorder, conservative treatments with antiepileptic drugs for seizures, trihexyphenidyl for dystonia, and thoracic surgery for pneumothorax and bleb resection were performed.

## 4 CONCLUSION

Although *FARSB* gene mutations are associated with a wide and variable spectrum of multisystem dysfunctions, they require awareness in clinical practice. Early diagnosis of this problem in atypical cases of multisystem disorders provides the chance to imply novel treatment approaches in order to control the burden, therefore performing genetic test, especially high‐throughput tests such as Whole Exome Sequencing could help us in the management of these patients.

## AUTHOR CONTRIBUTIONS

Parvaneh Karimzadeh: Diagnosing and referring the patients and revising the manuscript. Sepideh Rezakhani: Writing the manuscript and corresponding author. Mohammad Miryounesi: Performing and interpretation of the whole exom sequencing and revising the manuscript. Sahar Alijanpour: Assistance in performing and interpretation of the whole exom sequencing.

## CONFLICT OF INTEREST

Authors declare that they have no conflict of interest.

## CONSENT

An informed signed consent was obtained from the mother of the patients.

## Data Availability

Data available on request from the authors.

## References

[1] Kim MJ , Park B‐J , Kang Y‐S , et al. Downregulation of FUSE‐binding protein and c‐myc by tRNA synthetase cofactor p38 is required for lung cell differentiation. Nat Genet. 2003;34:330‐336. 12819782 10.1038/ng1182

[2] Ni R , Luo L . A noncanonical function of histidyl‐tRNA synthetase: inhibition of vascular hyperbranching during zebrafish development. FEBS Open Bio. 2018;8:722‐731. 10.1002/2211-5463.12420 PMC5929932 29744287

[3] Oprescu SN , Griffin LB , Beg AA , Antonellis A . Predicting the pathogenicity of aminoacyl‐tRNA synthetase mutations. Methods. 2017;113:139‐151. 27876679 10.1016/j.ymeth.2016.11.013 PMC5253330

[4] Wang Y , Zhou X‐L , Ruan Z‐R , Liu R‐J , Eriani G , Wang E‐D . A human disease‐causing point mutation in mitochondrial threonyl‐tRNA synthetase induces both structural and functional defects. J Biol Chem. 2016;291:6507‐6520. 26811336 10.1074/jbc.M115.700849 PMC4813579

[5] Finarov I , Moor N , Kessler N , Klipcan L , Safro MG . Structure of human cytosolic phenylalanyl‐tRNA synthetase: evidence for kingdom‐specific design of the active sites and tRNA binding patterns. Structure. 2010;18:343‐353. 20223217 10.1016/j.str.2010.01.002

[6] Jako E , Ittzes P , Szenes A , Kun A , Szathmáry E , Pal G . In silico detection of tRNA sequence features characteristic to aminoacyl‐tRNA synthetase class membership. Nucleic Acids Res. 2007;35:5593‐5609. 17704131 10.1093/nar/gkm598 PMC2018626

[7] Antonellis A , Oprescu SN , Griffin LB , Heider A , Amalfitano A , Innis JW . Compound heterozygosity for loss‐of‐function *FARSB* variants in a patient with classic features of recessive aminoacyl‐tRNA synthetase‐related disease. HumMmutat. 2018;39:834‐840. 10.1002/humu.23424 PMC5992071 29573043

[8] Miller SA , Dykes DD , Polesky HF . A simple salting out procedure for extracting DNA from human nucleated cells. Nucleic Acids Res. 1988;16:1215. 3344216 10.1093/nar/16.3.1215 PMC334765

[9] Li H , Durbin R . Fast and accurate long‐read alignment with burrows‐wheeler transform. Bioinformatics. 2010;26:589‐595. 20080505 10.1093/bioinformatics/btp698 PMC2828108

[10] Li H , Handsaker B , Wysoker A , et al. The sequence alignment/map format and SAMtools. Bioinformatics. 2009;25:2078‐2079. 19505943 10.1093/bioinformatics/btp352 PMC2723002

[11] McKenna A , Hanna M , Banks E , et al. The genome analysis toolkit: a MapReduce framework for analyzing next‐generation DNA sequencing data. Genome Res. 2010;20:1297‐1303. 20644199 10.1101/gr.107524.110 PMC2928508

[12] Antonellis A , Green ED . The role of aminoacyl‐tRNA synthetases in genetic diseases. Annu Rev Genomics Hum Genet. 2008;9:87‐107. 18767960 10.1146/annurev.genom.9.081307.164204

[13] Meyer‐Schuman R , Antonellis A . Emerging mechanisms of aminoacyl‐tRNA synthetase mutations in recessive and dominant human disease. Hum Mol Genet. 2017;26:R114‐R127. 28633377 10.1093/hmg/ddx231 PMC5886470

[14] Fuchs SA , Schene IF , Kok G , et al. Aminoacyl‐tRNA synthetase deficiencies in search of common themes. Genet Med. 2019;21:319‐330. 29875423 10.1038/s41436-018-0048-y PMC7091658

[15] Krenke K , Szczałuba K , Bielecka T , et al. *FARSA* mutations mimic phenylalanyl‐tRNA synthetase deficiency caused by *FARSB* defects. Clin Genet. 2019;96:468‐472. 31355908 10.1111/cge.13614

[16] Zadjali F , Al‐Yahyaee A , Al‐Nabhani M , et al. Homozygosity for *FARSB* mutation leads to Phe‐tRNA synthetase‐related disease of growth restriction, brain calcification, and interstitial lung disease. Hum Mutat. 2018;39:1355‐1359. 30014610 10.1002/humu.23595

[17] Schuch LA , Forstner M , Rapp CK , et al. *FARS1* ‐related disorders caused by bi‐allelic mutations in cytosolic phenylalanyl‐tRNA synthetase genes: look beyond the lungs! Clin Genet. 2021;99:789‐801. 33598926 10.1111/cge.13943

[18] Kok G , Tseng L , Schene IF , et al. Treatment of ARS deficiencies with specific amino acids. Genet Med. 2021;23:2202‐2207. 34194004 10.1038/s41436-021-01249-z PMC8244667

